# Hematoma volume and other prognostic factors with mortality in spontaneous intracerebral hemorrhage

**DOI:** 10.1186/2197-425X-3-S1-A981

**Published:** 2015-10-01

**Authors:** HB Rotzel, A Serrano Lázaro, D Aguillón Prada, A Mesejo Arizmendi, C Sanchís Piqueras, M Rodriguez Gimillo, ML Blasco Cortés, MN Carbonell Monleón, O Díaz Barina

**Affiliations:** Hospital Clinic Universitario de Valencia, Valencia, Spain

## Introduction

Intracerebral hemorrhage (ICH), a subtype of stroke associated with high mortality and the majority of survivors will remain permanently disabled. Identifying prognostic factors will determine the severity and guide the treatment for different patients.

## Objective

To relate the volume of hematoma and other prognostic factors with mortality in patients with spontaneous intracerebral hemorrhage (ICH).

## Material and Methods

All patients admitted to the ICU with the diagnosis of spontaneous ICH were collected. We determine the hematoma volume at admission with the modified formula Kothari (AxBxC / 2 cc), the location of hemastom as posterior fossa or supratentorial, severity scales (GCS at admission, SOFA, APACHE II), medical history and complications during the ICU admission. Used percentils (%,) mean (SD) and median (min / max). For the univariate analysis we used U-Mann Whitney and Chi square with p < 0.05 significant. Subsequently we performed a multivariate analysis using binary regression (OR with 95% CI) and significant p < 0.05.

## Results

101 patients were included. 66% men with a mean age 61.8 ± 12.7 years. Our overall mortality was 35%. 82% were supratentorial and 16% infratentorial. The median APACHE II 13 (0-30), SOFA 4 (0-14), GCS 12 (3-15) and mean volume of 37.8 cc hematoma. In the univariate analysis was significantly correlated with mortality: GCS at admission (p 0.001), APACHE II (p 0.001) and SOFA (p 0.000), taking oral anticoagulants (ACOs) (p 0.05), LOS in hospital (p 0.05) and complications as intracranial hypertension (p 0.000), rebleeding (0.01), herniation (p 0.000) and eadema (± 0,001). After the multivariate analysis, only de volume of hematoma (OR 1.2, 95% CI 1.1-1.2; p 0.01), intracranial hypertension in the first 48 h (OR 3 5; 95% CI 1.12 to 10.99; p 0.03), herniation (OR 7.08, 95% CI 2.77 to 18.09; p 0.000) and SOFA (OR 1.35; 95 % 1.16 to 1.57; p 0.000). was correlated significant with mortality. As for the volume of hematoma we determied a cuttoff point > 20cc obtaining statistical significance in its relation with mortality (p 0.02. In a subgroup analysis between posterior fossa and supratentorial with volumes> 15 cc and 45 cc respectively we obtained also statistically significance with mortality (p 0.037 and 0.038 respectively).

## Conclusions

SOFA, volumen of hematoma at admission, intracranial hypertension and herniation are related to mortality in patients with spontaneous intracerebral hemorrhage.

## References

[1] Specogna AV, Turin TC, Patten SB, Hill MD (2014). Factors associated with early deterioration after spontaneous intracerebral hemorrhage: a systematic review and meta-analysis. PLoS One.

[2] Gupta M, Verma R, Parihar A, Garg RK, Singh MK, Malhotra HS (2014). Perihematomal edema as predictor of outcome in spontaneous intracerebral hemorrhage. J Neurosci Rural Pract.

